# Synergism of MSC-secreted HGF and VEGF in stabilising endothelial barrier function upon lipopolysaccharide stimulation via the Rac1 pathway

**DOI:** 10.1186/s13287-015-0257-0

**Published:** 2015-12-16

**Authors:** Yi Yang, Qi-hong Chen, Ai-ran Liu, Xiu-ping Xu, Ji-bin Han, Hai-bo Qiu

**Affiliations:** Department of Critical Care Medicine, Zhong-Da Hospital, School of Medicine, Southeast University, 87 Dingjiaqiao Road, Nanjing, 210009 People’s Republic of China

**Keywords:** Mesenchymal stem cells, Vascular endothelial growth factor, Hepatocyte growth factor, Endothelial permeability, Acute lung injury

## Abstract

**Background:**

Mesenchymal stem cells (MSCs) stabilise endothelial barrier function in acute lung injury via paracrine hepatocyte growth factor (HGF). Vascular endothelial growth factor (VEGF), which is secreted by MSCs, is another key regulator of endothelial permeability; however, its role in adjusting permeability remains controversial. In addition, whether an interaction occurs between HGF and VEGF, which are secreted by MSCs, is not completely understood.

**Methods:**

We introduced a co-cultured model of human pulmonary microvascular endothelial cells (HPMECs) and MSC conditioned medium (CM) collected from MSCs after 24 h of hypoxic culture. The presence of VEGF and HGF in the MSC-CM was neutralised by anti-VEGF and anti-HGF antibodies, respectively. To determine the roles and mechanisms of MSC-secreted HGF and VEGF, we employed recombinant humanised HGF and recombinant humanised VEGF to co-culture with HPMECs. Additionally, we employed the RhoA inhibitor C3 transferase and the Rac1 inhibitor NSC23766 to inhibit the activities of RhoA and Rac1 in HPMECs treated with MSC-CM or VEGF/HGF with the same dosage as in the MSC-CM. Then, endothelial paracellular and transcellular permeability was detected. VE-cadherin, occludin and caveolin-1 protein expression in HPMECs was measured by western blot. Adherens junction proteins, including F-actin and VE-cadherin, were detected by immunofluorescence.

**Results:**

MSC-CM treatment significantly decreased lipopolysaccharide-induced endothelial paracellular and transcellular permeability, which was significantly inhibited by pretreatment with HGF antibody or with both VEGF and HGF antibodies. Furthermore, MSC-CM treatment increased the expression of the endothelial intercellular adherence junction proteins VE-cadherin and occludin and decreased the expression of caveolin-1 protein. MSC-CM treatment also decreased endothelial apoptosis and induced endothelial cell proliferation; however, the effects of MSC-CM treatment were inhibited by pretreatment with HGF antibody or with both HGF and VEGF antibodies. Additionally, the effects of MSC-CM and VEGF/HGF on reducing endothelial paracellular and transcellular permeability were weakened when HPMECs were pretreated with the Rac1 inhibitor NSC23766.

**Conclusion:**

HGF secreted by MSCs protects the endothelial barrier function; however, VEGF secreted by MSCs may synergize with HGF to stabilise endothelial cell barrier function. Rac1 is the pathway by which MSC-secreted VEGF and HGF regulate endothelial permeability.

## Background

Acute lung injury (ALI) is characterised by increased lung permeability, pulmonary oedema and diffuse inflammation and is involved in the disruption of alveolar–capillary membranes [1]. Many agents, such as bacterial lipopolysaccharide (LPS), lead to an increase in permeability by activating the inflammatory response, which contributes to the development of ALI [2]. Because endothelial cells (ECs) play a major role in ALI by changing their barrier permeability, pulmonary EC dysfunction is a key component of ALI pathogenesis. Thus, stabilising EC barrier function is critical for treating ALI.

Our previous study provided convincing data regarding the beneficial effects of mesenchymal stem cells (MSCs) in treating endotoxin-induced ALI [3]. MSCs have potent effects on alleviating vascular endothelium injury by inhibiting endothelial permeability after injury via the modulation of adherens junction (AJ) proteins [4]. However, the detailed pathogenesis of MSCs in reducing endothelial permeability remains unclear. Studies have shown that the multipotent differentiation of MSCs contributes minimally to their beneficial effects, while paracrine activity may play a predominant role in MSC function [5, 6]. Thus, MSCs improve endothelial injury primarily through a paracrine mechanism.

MSC-secreted hepatocyte growth factor (HGF) and vascular endothelial growth factor (VEGF) are two important factors associated with endothelial permeability [7]. HGF is present in the lung circulation under pathological conditions such as ALI and exhibits sustained barrier protective effects on human pulmonary ECs [8]. VEGF increases paracellular endothelial permeability, in contrast to HGF, but decreases EC apoptosis and improves cell viability [9]. Furthermore, VEGF reduces the transcellular permeability of ECs upon LPS stimulation [10]. Different quantities and proportions of HGF and VEGF may exhibit different effects on endothelial permeability. Therefore, determining the detailed roles of MSC-secreted HGF and VEGF in regulating endothelial permeability is necessary. An LPS-induced increase in endothelial permeability is regulated by Rho GTPases. Rac1 is required for the maintenance of intercellular adherens and tight junctions; however, RhoA contributes to the breakage of intercellular adherens and tight junctions [11]. The role of Rho GTPases in HGF and VEGF regulation of endothelial permeability is also not fully understood.

The aim of the present study was to determine the effects and mechanisms of MSC-secreted HGF and VEGF on LPS-induced endothelial permeability. We investigated the effects of MSC-secreted HGF and VEGF on endothelial paracellular and transcellular permeability in in vitro co-culture experiments by neutralising HGF or VEGF with HGF or VEGF antibody and then explored the mechanisms by which MSC-secreted HGF and VEGF regulate endothelial permeability by inhibiting RhoA and Rac1 activities with RhoA and Rac1 inhibitors.

## Materials and methods

### Cell culture

Human mesenchymal stem cells (hMSCs) were purchased from Cyagen Biosciences, Inc. (Guangzhou, China). Human pulmonary microvascular endothelial cells (HPMECs) were obtained from ScienCell Research Laboratories. hMSCs were cultured in MSC growth medium (Cyagen Biosciences, Inc.), and HPMECs were cultured in endothelial growth medium (EGM-2; ScienCell Research Laboratories, USA). The cells were cultured in a humidified 5 % CO_2_ incubator at 37 °C. The culture media were changed every 2–3 days, and the cells at passages 3–7 were used for all experiments.

### Hypoxia culture

For hypoxia treatment, hMSCs were cultured for 3 days until confluent. A serum-free culture (supplemented with 0.05 % bovine serum albumin (BSA)) was used before hypoxic culture. hMSCs (1,000,000 cells per culture flask) were placed in a hypoxia incubator (BioSpherix, USA) for 24 h in an atmosphere of N_2_ (94.5 %), O_2_ (0.5 %) and CO_2_ (5 %). After 24 h of hypoxic culture, supernatants were collected and centrifuged to remove debris.

### Enzyme-linked immunosorbent assay

**Fig. 1:**
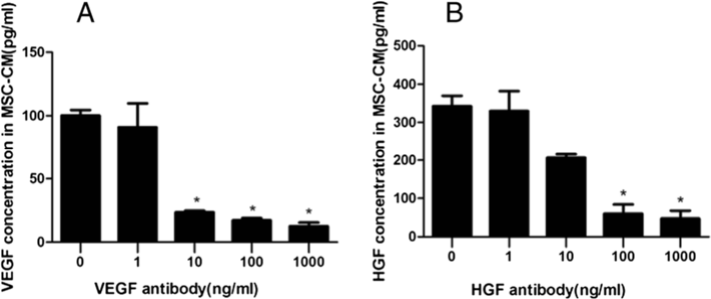
The blocking effect of **a** anti-VEGF and **b** anti-HGF antibody on VEGF and HGF in MSC-CM. The results showed the actual levels of HGF and VEGF were 343 pg/ml and 99 pg/ml, respectively, in MSC-CM after 24 h of hypoxic culture. However, VEGF in MSC-CM was significantly blocked by 10 ng/ml or more anti-VEGF antibody (*p* < 0.01), while 100 ng/ml or more anti-HGF antibody significantly blocked HGF in MSC-CM (*p* < 0.01); n = 3, *p < 0.01 vs. 0 ng/ml. *CM* Conditioned medium, *HGF* Hepatocyte growth factor, *MSC* Mesenchymal stem cell, *VEGF* Vascular endothelial growth factor

After 24 h of hypoxic culture, supernatants were collected and centrifuged to remove debris. VEGF and HGF were determined via an enzyme-linked immunosorbent assay (ELISA) using commercially available ELISA sets (ExCell Biology, Inc., Shanghai, China). We further tested VEGF and HGF concentration by ELISA quantification after adding anti-VEGF and anti-HGF antibody on MSC conditioned medium (CM) using 1 ng/ml, 10 ng/ml, 100 ng/ml, and 1000 ng/ml, respectively. ELISA was performed according to the manufacturer’s instructions. All samples were measured in duplicate. We have assessed the blocking effect of anti-HGF and anti-VEGF antibody on MSC-CM. The result showed the actual levels of HGF and VEGF were 343 pg/ml and 99 pg/ml, respectively, in MSC-CM after 24 h of hypoxic culture. However, VEGF in MSC-CM was significantly blocked by 10 ng/ml or more anti-VEGF antibody (*p* < 0.01), while 100 ng/ml or more anti-HGF antibody significantly blocked HGF in MSC-CM (*p* < 0.01) (Fig. 1).

### Co-culture protocol

HPMECs were cultured at a density of 50,000 cells per well in six-well culture plates. After the HPMECs reached confluence, the medium was changed with fresh culture medium or hMSC hypoxia culture supernatants that contained anti-HGF antibody (100 ng/ml; Abcam, Hong Kong), anti-VEGF antibody (10 ng/ml; Abcam, Hong Kong), or both anti-HGF (100 ng/ml) and anti-VEGF antibodies (10 ng/ml). After the HPMECs were cultured for 24 h, HPMEC monolayers were treated with 100 ng/ml LPS (Sigma, USA). To determine the roles and mechanisms of MSC-secreted HGF and VEGF, we employed HPMECs with LPS stimulation to co-culture with recombinant humanised HGF (343 pg/ml; PEPROPECH, USA) and recombinant humanised VEGF (99 pg/ml; PEPROPECH, USA) with the same dosage as in the MSC-CM. Furthermore, the RhoA inhibitor C3 transferase (5 μg/ml; Cytoskeleton, USA) and the Rac1 inhibitor NSC23766 (50 μM; TOCRIS, USA) were used to inhibit the activities of RhoA and Rac1 in HPMECs treated with MSC-CM or recombinant VEGF/HGF (99 pg/ml and 343 pg/ml, respectively).

### HPMEC permeability examination

HPMECs were seeded at 50,000 cells per insert well (0.4 μm pore size polyester membrane from Corning, Inc., USA) and cultured for 1 to 3 days to allow the growth of a confluent monolayer. After different groups received different treatments, HPMEC monolayers were treated with 100 ng/ml LPS for 6 h before testing permeability. Paracellular and transcellular permeability was tested as described previously [12]. In brief, paracellular permeability was tested by adding 10 μl of 10 mg/ml fluorescein isothiocyanate (FITC)-Dextran (Sigma-Aldrich) to the upper chamber. The FITC-Dextran component from samples was obtained 40 min after the addition of FITC-Dextran. Medium (100 μl) was withdrawn from the lower well and the upper well, respectively. Then measurements were taken with a microplate reader using excitation and emission wavelengths of 490 and 525 nm, respectively. Paracellular permeability was calculated as previously described [12]. To test transcellular permeability, 10 μl of 0.4 mg/ml FITC-BSA (Invitrogen, USA) was added to the upper chamber. The remaining experimental procedure of transcellular permeability detection is the same as that of paracelluar permeability.

### Western blot analysis

After treatment, total protein from HPMECs was extracted using RIPA lysis buffer supplemented with 1 mmol/l phenylmethanesulfonyl fluoride (Beyotime Institute of Biotechnology), followed by separation by 6 or 12 % SDS-PAGE and transfer onto polyvinylidene fluoride membranes (Nanjing, China). Then the membranes were blocked in phosphate-buffered saline-Tween (PBS-T) containing 5 % milk for 2 h at room temperature and incubated at 4 °C overnight with primary antibodies against VE-cadherin (1:1000; Cell Signaling), occludin (1:250; Abcam) or caveolin-1 (1:1000; Epitomics). The next day, the membranes were washed in PBS-T and incubated in peroxidase-conjugated secondary antibody (1:1000; HuaAn Biotechnology, Hangzhou, China) for 1 h at room temperature. Signals from immunoreactive bands were visualised using a chemiluminescence imaging system (ChemiQ 4800 mini, Ouxiang, Shanghai, China) after incubation with a horseradish peroxidase.

### Immunofluorescence

In total, 5 × 10^4^ HPMECs were seeded in six-well culture plates and cultured for 1 to 3 days to allow for the growth of a confluent monolayer. After treatment, HPMEC monolayers were treated with 100 ng/ml LPS before immunofluorescence analysis. After the HPMECs were treated for 6 h, they were washed in cold PBS and fixed in 4 % paraformaldehyde. Then the cells were incubated with 1 % BSA in PBS for 30 min to block non-specific binding and incubated with VE-cadherin antibody (1:50; Cell Signaling) or F-actin antibody (5 μg/ml; Abcam) at 4 °C overnight. Next, the cells were incubated with FITC-conjugated goat anti-mouse IgG (1:200; Jackson). Finally, DAPI (1:100; 4,6-diamidino-2-phenylindole) was used to stain nuclei. Single plain images of cells were obtained by fluorescence microscopy (Olympus).

### Pull-down assays

HPMECs were cultured in six-well culture plates until reaching confluence and then incubated overnight in medium supplemented with 1 % serum before treatment. RhoA activity was measured using a recombinant GST-Rho binding domain bound to glutathione beads (Thermo Scientific), and Rac1 activity was measured using GST-human Pak1-PBD (Thermo Scientific). Affinity-precipitated RhoA and Rac1 proteins were separated by SDS-PAGE and detected by western blot. The detailed protocols for RhoA and Rac1 pull-downs were provided by Thermo Scientific (RhoA and Rac1 activation assay kits).

### Viability and apoptosis assays

The viability of HPMECs was evaluated by 3-(4,5-dimethylthiazol-2-yl)-2,5-diphenyltetrazolium (MTT; Sigma) assay. HPMECs were cultured in 24-well culture plates until reaching 75–85 % confluence. After the cells were treated, 60 μl MTT (5 mg/ml) was added to each well and incubated at 37 °C for 4 h. Then 200 μl dimethyl sulfoxide (DMSO; Sigma) was added to the wells and incubated for 15 min. Finally, HPMEC viability was assessed by measuring the absorbances of the sample at 570 nm and 630 nm.

HPMEC apoptosis was assessed using an annexin V-FITC assay kit (Sigma) according to the manufacturer’s instructions. After treatment, HPMECs were harvested and washed in PBS. HPMECs were suspended in PBS at a concentration of 1 × 10^6^ cells/ml and then incubated with 5 μl annexin V-FITC conjugate (annexin V) and 10 μl propidium iodide solution, followed by analysis using a flow cytometer (BD Biosciences).

### Statistical analyses

Statistical analyses were performed using the SPSS 16.0 software package. The data were presented as the mean ± standard deviation. For group comparisons, one-way analysis of variance was used, followed by Tukey’s multiple comparison tests. *p* values less than 0.05 were considered statistically significant.

## Results

### MSC-secreted HGF and VEGF have a synergistic effect on reducing HPMEC permeability

**Fig. 2:**
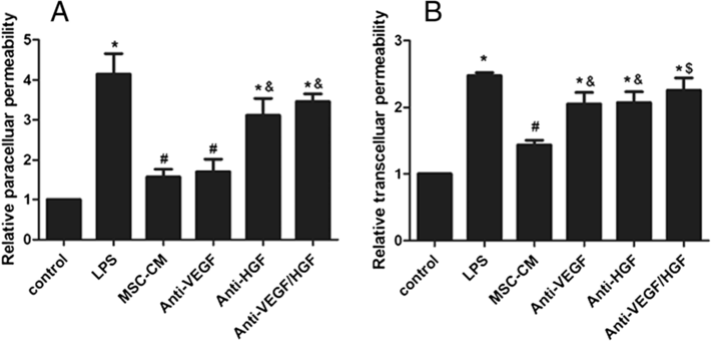
MSCs reduced HPMEC permeability by secreting paracrine VEGF and HGF. The result showed that MSC-CM treatment reduced LPS-induced endothelial paracellular permeability; however, the effect of MSC-CM treatment was significantly blocked by anti-HGF antibody (*p* < 0.05 and *p* < 0.01; **a**). Meanwhile, MSC-CM treatment reduced LPS-induced transcellular permeability (**b**), and the effect of MSC-CM treatment was significantly blocked by anti-HGF antibody or anti-VEGF antibody. Furthermore, the role of MSCs in reducing transcellular permeability was clearly inhibited by anti-HGF and anti-VEGF antibodies. Adding MSC-CM in all groups except control group and LPS group. n = 3, *p < 0.01 vs control group; ^#^ *p* < 0.01 vs LPS group; &*p* < 0.05; ^$^ *p* < 0.01 vs MSC-CM group. *CM* Conditioned medium, *HGF* Hepatocyte growth factor, *LPS* Lipopolysaccharide, *MSC* Mesenchymal stem cell, *VEGF* Vascular endothelial growth factor

To evaluate the effects of MSC-secreted HGF and VEGF on HPMEC permeability, we introduced a co-cultured model using HPMECs and MSC-CM collected from MSCs after 24 h hypoxia culture. VEGF and HGF in the MSC-CM were neutralised with anti-VEGF and HGF antibodies, respectively, followed by the detection of endothelial paracellular and transcellular permeability. The results showed that MSC-CM treatment reduced LPS-induced endothelial paracellular permeability; however, the MSC-CM effect was significantly blocked by anti-HGF antibody (*p* < 0.05 and *p* < 0.01; Fig. 2a). Meanwhile, MSC-CM treatment reduced LPS-induced transcellular permeability, and the effect of MSC-CM treatment was significantly blocked by anti-HGF antibody or anti-VEGF antibody. Furthermore, the role of MSCs in reducing transcellular permeability was clearly inhibited by anti-HGF and anti-VEGF antibodies (*p* < 0.05; Fig. 2b). The results suggested that MSCs lessen endothelial paracellular permeability by secreting paracrine HGF and reduce transcellular permeability by secreting paracrine VEGF and HGF.

### The combined effects of MSC-secreted HGF and VEGF on endothelial adhesive junction upregulation and caveolin-1 protein expression downregulation

**Fig. 3:**
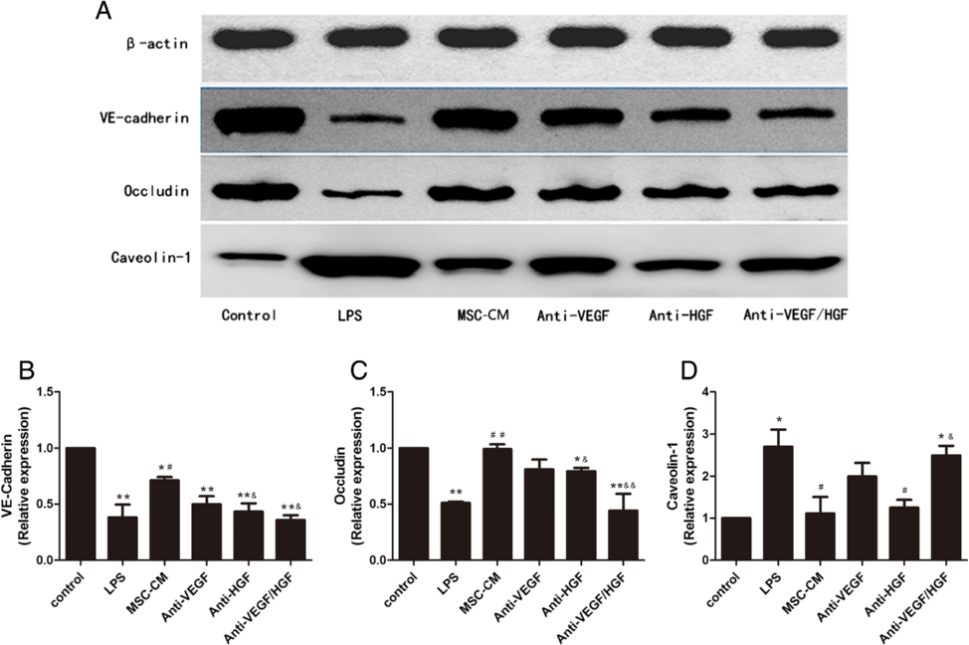
MSC-secreted paracrine HGF upregulated endothelial VE-cadherin protein expression and decreased caveolin-1 protein expression. The results showed that LPS stimulation of HPMECs reduced the expression of VE-cadherin and occludin protein (*p* < 0.01; **a**, **b**, **c**) but increased the expression of caveolin-1 protein (*p* < 0.05; **a**, **d**) and that these effects were inhibited by MSCs. However, the effect of MSCs was significantly blocked by anti-HGF antibody (*p* < 0.05). Furthermore, the role of MSCs in reducing caveolin-1 protein expression was clearly inhibited by anti-HGF and anti-VEGF antibodies. Adding MSC-CM in all groups except control group and LPS group. n = 3, *p < 0.05, **p < 0.01 vs. control group; ^#^ *p* < 0.05 vs. LPS group; &*p* < 0.05 vs. MSC-CM group. *CM* Conditioned medium, *HGF* Hepatocyte growth factor, *LPS* Lipopolysaccharide, *MSC* Mesenchymal stem cell, *VEGF* Vascular endothelial growth factor

To illustrate the effects of MSC-secreted HGF and VEGF on endothelial permeability-associated proteins, we examined endothelial VE-cadherin, occludin and caveolin-1 protein expression under co-culture conditions. The results showed that LPS stimulation of HPMECs reduced the expression of VE-cadherin and occludin proteins (*p* < 0.01; Fig. 3b and c) and increased the expression of caveolin-1 protein (*p* < 0.05; Fig. 3d) and that these effects were inhibited by MSC. However, the effect of MSCs was significantly blocked by anti-HGF antibody (*p* < 0.05; Fig. 3). Furthermore, the role of MSCs in reducing caveolin-1 protein expression was clearly inhibited by anti-HGF and anti-VEGF antibodies. The results indicated that MSC-secreted HGF upregulated endothelial VE-cadherin and occludin protein expression and downregulated caveolin-1 protein expression.

### MSC-secreted HGF restored endothelial VE-cadherin and F-actin remodelling

**Fig. 4:**
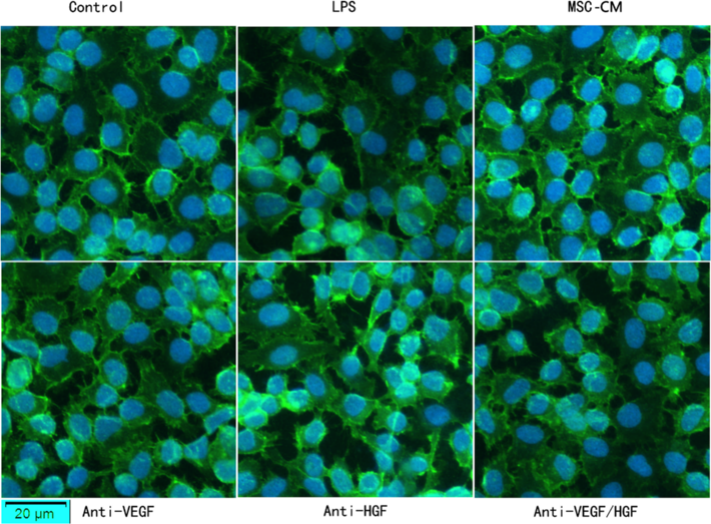
MSC-secreted HGF restored endothelial VE-cadherin remodelling. LPS causes the remodelling of the junctional localisation of VE-cadherin, which causes HPMEC to contract, increasing paracellular permeability. After 24 h of MSC-CM and HPMEC co-culture, the remodelling of the junctional localisation of VE-cadherin was partially restored. However, neutralising HGF from the MSC-CM with anti-HGF antibody caused VE-cadherin to be disrupted again. Adding MSC-CM in all groups except control group and LPS group. *CM* Conditioned medium, *HGF* Hepatocyte growth factor, *LPS* Lipopolysaccharide, *MSC* Mesenchymal stem cell, *VEGF* Vascular endothelial growth factor

**Fig. 5:**
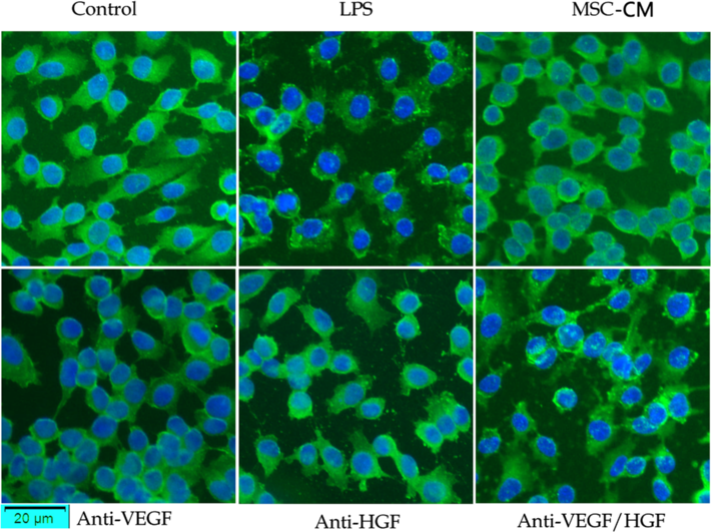
MSC-secreted HGF restored endothelial F-actin remodelling. LPS cause “actin stress fibre” formation in HPMEC, which is the movement of actin from the cortical rim to a disorganized intracellular fashion. It is this stress fibre formation which causes the cells to contract, increasing paracellular permeability. After 24 h of MSC-CM and HPMEC co-culture, actin stress fibre was partially restored. However, neutralising HGF from the MSC-CM with anti-HGF antibody caused actin stress fibre formation again. Adding MSC-CM in all groups except control group and LPS group. *CM* Conditioned medium, *HGF* Hepatocyte growth factor, *LPS* Lipopolysaccharide, *MSC* Mesenchymal stem cell, *VEGF* Vascular endothelial growth factor

We further investigated the role of MSC-secreted paracrine VEGF and HGF in regulating the remodelling of the endothelial actin cytoskeleton and intercellular AJs. LPS causes the remodelling of the junctional localisation of VE-cadherin, which causes HPMEC to contract, increasing paracellular permeability. After 24 h of MSC-CM and HPMEC co-culture, the remodelling of the junctional localisation of VE-cadherin was partially restored. However, neutralising HGF from the MSC-CM with anti-HGF antibody caused VE-cadherin to be disrupted again (Fig. 4). Furthermore LPS causes “actin stress fibre” formation in HPMEC, which is the movement of actin from the cortical rim to a disorganized intracellular fashion. It is this stress fibre formation which causes the cells to contract, increasing paracellular permeability. After 24 h of MSC-CM and HPMEC co-culture, actin stress fibre was partially restored. However, neutralising HGF from the MSC-CM with anti-HGF antibody caused actin stress fibre formation again (Fig.5).

**Fig. 6:**
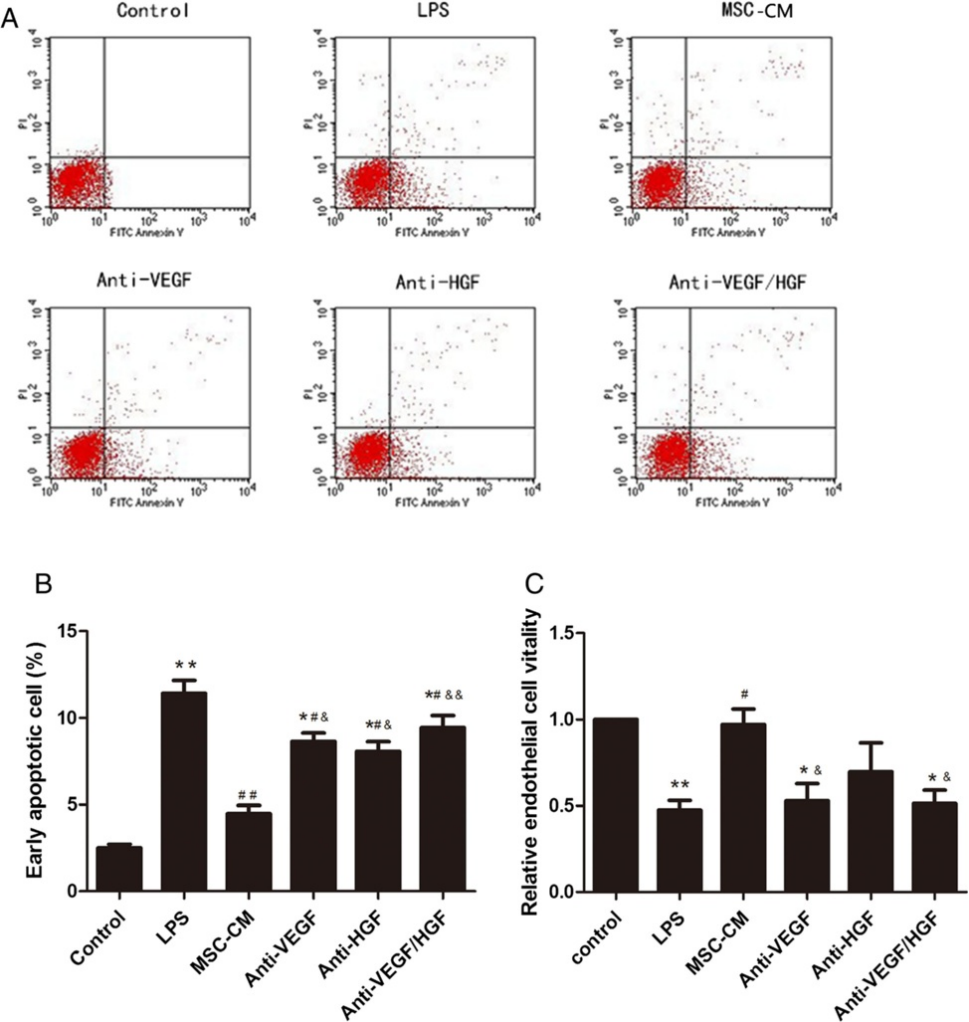
MSC-secreted VEGF and HGF decreased HPMEC apoptosis and improved cell viability. **a**, **b** LPS induced early apoptosis of HPMECs. MSC-CM treatment significantly reduced the number of early apoptotic cells. However, the effect of MSCs was significantly blocked by neutralising HGF or VEGF from the MSC-CM with anti-HGF or anti-VEGF antibody, respectively. **c** MTT assay confirmed that MSC-CM treatment restored cell viability to a greater extent than LPS stimulation only. Additionally, the effect of MSC-CM treatment was significantly inhibited by neutralising VEGF from the MSC-CM with anti-VEGF antibody. Adding MSC-CM in all groups except control group and LPS group. n = 3, *p < 0.05, **p < 0.01 vs. control group; ^#^ *p* < 0.05 vs. LPS group; &*p* < 0.05; &&*p* < 0.01 vs. MSC-CM group. *CM* Conditioned medium, *FITC* Fluorescein isothiocyanate, *HGF* Hepatocyte growth factor, *LPS* Lipopolysaccharide, *MSC* Mesenchymal stem cell, *VEGF* Vascular endothelial growth factor

### MSC-secreted VEGF and HGF decreased HPMEC apoptosis and improved cell viability

HPMEC survival was evaluated by apoptosis and cell viability assays. The effect of MSCs on HPMEC apoptosis was assessed using an annexin V-FITC assay kit. The results showed that LPS induced early apoptosis of HPMECs. MSC-CM treatment significantly reduced the number of early apoptotic cells (*p* < 0.05; Fig. 6a and b). However, the effect of MSCs was significantly blocked by neutralising HGF or VEGF from the MSC-CM with anti-HGF or anti-VEGF antibody, respectively (*p* < 0.05 and *p* < 0.01, respectively; Fig. 6a and b). The cell viability results of the MTT assay confirmed that MSC-CM restored cell viability to a greater extent than did LPS stimulation only (*p* < 0.05; Fig. 6c). Additionally, the effect of MSC-CM treatment was significantly inhibited by neutralising VEGF from the MSC-CM with anti-VEGF antibody (*p* < 0.05; Fig. 6c).

### MSC-secreted VEGF and HGF upregulated Rac1 activity and downregulated RhoA activity in LPS-stimulated HPMECs

**Fig. 7:**
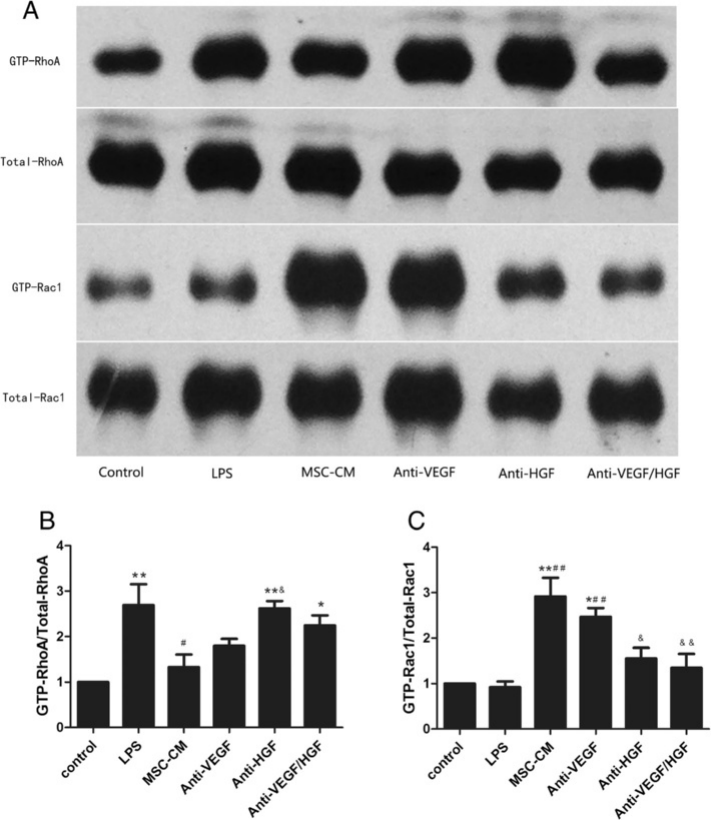
MSC-secreted VEGF and HGF upregulated Rac1 activity (Fig. 7a and b) and downregulated RhoA activity (Fig.7a and c) in LPS-stimulated HPMECs. MSC-CM treatment upregulated Rac1 activity and downregulated RhoA activity in LPS-stimulated HPMECs. However, the effect of MSCs was significantly inhibited by neutralising HGF or both VEGF and HGF from the MSC-CM with HGF antibody or both VEGF and HGF antibodies. Neutralising VEGF from the MSC-CM with anti-VEGF did not affect the effect of MSCs. Adding MSC-CM in all groups except control group and LPS group. n = 3, *p < 0.05; **p < 0.01 vs. control group; ^#^ *p* < 0.05 vs. LPS group; &*p* < 0.05; &&*p* < 0.01 vs. MSC-CM group. *CM* Conditioned medium, *HGF* Hepatocyte growth factor, *LPS* Lipopolysaccharide, *MSC* Mesenchymal stem cell, *VEGF* Vascular endothelial growth factor

To explore the mechanisms by which MSC-secreted HGF and VEGF improved HPMEC permeability, we investigated the effects of MSC-secreted HGF and VEGF on Rac1 and RhoA activities in LPS-stimulated HPMECs. Our results showed that MSC-CM treatment upregulated Rac1 activity and downregulated RhoA activity in LPS-stimulated HPMECs. However, the effect of MSCs was significantly inhibited by neutralising HGF or both VEGF and HGF from the MSC-CM with HGF antibody or both VEGF and HGF antibodies (*p* < 0.05 and *p* < 0.01, respectively; Fig. 7). Neutralising VEGF from the MSC-CM with anti-VEGF did not affect the effect of MSCs. These result suggested that MSC-secreted HGF upregulated Rac1 activity and downregulated RhoA activity in LPS-stimulated HPMECs.

### RhoA and Rac1 activities in LPS-stimulated HPMECs were inhibited by RhoA and Rac1 inhibitors, respectively

**Fig. 8:**
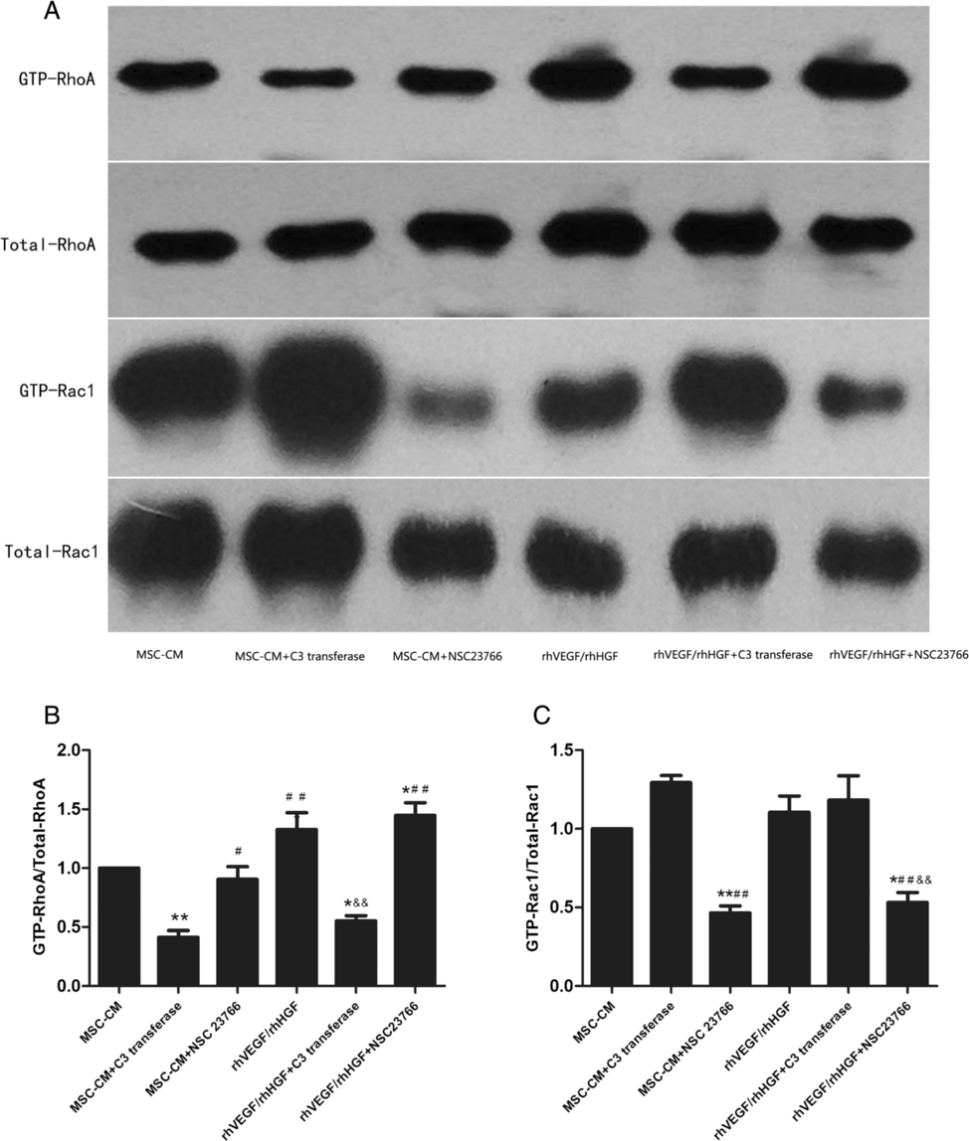
RhoA and Rac1 activities in LPS-stimulated HPMECs were restrained by the RhoA and Rac1 inhibitors, respectively. The results showed that C3 transferase significantly inhibited RhoA activity (Fig. 8a and b) and that NSC23766 inhibited Rac1 activity (Fig. 8a and c) in injured HPMECs treated with MSCs or VEGF/HGF. n = 3, *p < 0.05; **p < 0.01 vs. MSC group; ^#^ *p* < 0.05 vs. LPS group; &*p* < 0.05; &&*p* < 0.01 vs. VEGF/HGF group. *CM* Conditioned medium, *HGF* Hepatocyte growth factor, *LPS* Lipopolysaccharide, *MSC* Mesenchymal stem cell, *VEGF* Vascular endothelial growth factor

The above research suggested that the mechanism by which MSC-secreted VEGF and HGF reduced HPMEC permeability might function via regulating RhoA and Rac1 activities. To confirm this hypothesis, we employed the RhoA inhibitor C3 transferase and the Rac1 inhibitor NSC23766 to inhibit RhoA and Rac1 activities, respectively, in HPMECs treated with MSCs or VEGF/HGF. Furthermore, we adopted human recombinant VEGF and HGF with the same dosage as in the MSC-CM to culture HPMECs. The results showed that C3 transferase significantly inhibited RhoA activity and that NSC23766 inhibited Rac1 activity in injured HPMECs treated with MSCs or VEGF/HGF (*p* < 0.05 and *p* < 0.01, respectively; Fig. 8).

### MSC-secreted VEGF and HGF reduced LPS-stimulated HPMEC permeability by upregulating Rac1 activity in HPMECs

**Fig. 9:**
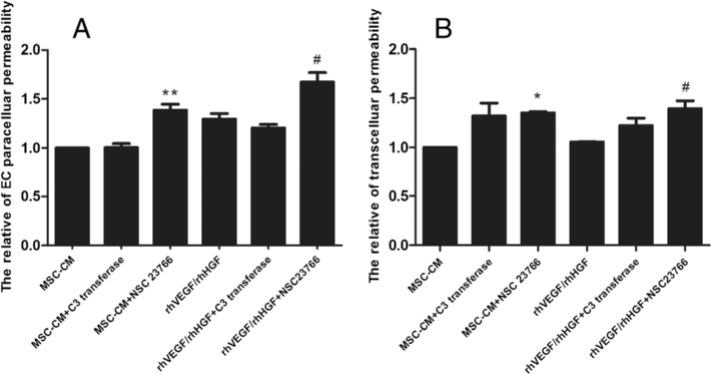
MSC-secreted VEGF and HGF reduced LPS-stimulated HPMEC permeability by upregulating Rac1 activity in HPMECs. Our research showed that the effect of MSCs on reducing endothelial **a** paracellular and **b** transcellular permeability was weakened when injuried HPMECs were pretreated with the Rac1 inhibitor NSC23766. Similarly, the roles of VEGF and HGF in improving endothelial permeability were significantly inhibited by NSC23766 pretreatment. n = 3, *p < 0.05, *p < 0.01 vs. MSC group; ^#^ *p* < 0.05 vs. LPS group and vs. VEGF/HGF group. *CM* Conditioned medium, *EC* Endothelial cell, *HGF* Hepatocyte growth factor, *LPS* Lipopolysaccharide, *MSC* Mesenchymal stem cell, *VEGF* Vascular endothelial growth factor

To explore the mechanism by which MSC-secreted VEGF and HGF improve HPMEC permeability, we further investigated the effects of MSC and VEGF/HGF on C3 transferase- and NSC23766-inhibited endothelial paracellular and transcellular permeability. Our research showed that the effect of MSCs on reducing endothelial paracellular and transcellular permeability was weakened when HPMECs were pretreated with the Rac1 inhibitor NSC23766. Similarly, the roles of VEGF and HGF in improving endothelial permeability were significantly inhibited by NSC23766 pretreatment (*p* < 0.05 and *p* < 0.01, respectively; Fig. 9). Our research indicated that MSC-secreted VEGF and HGF reduced injured HPMEC permeability by upregulating Rac1 activity in HPMECs.

### VEGF/HGF and MSC treatments enhanced VE-cadherin and occludin protein expression and reduced caveolin-1 protein expression in LPS-stimulated HPMECs via the RhoA/Rac1 pathway

**Fig. 10:**
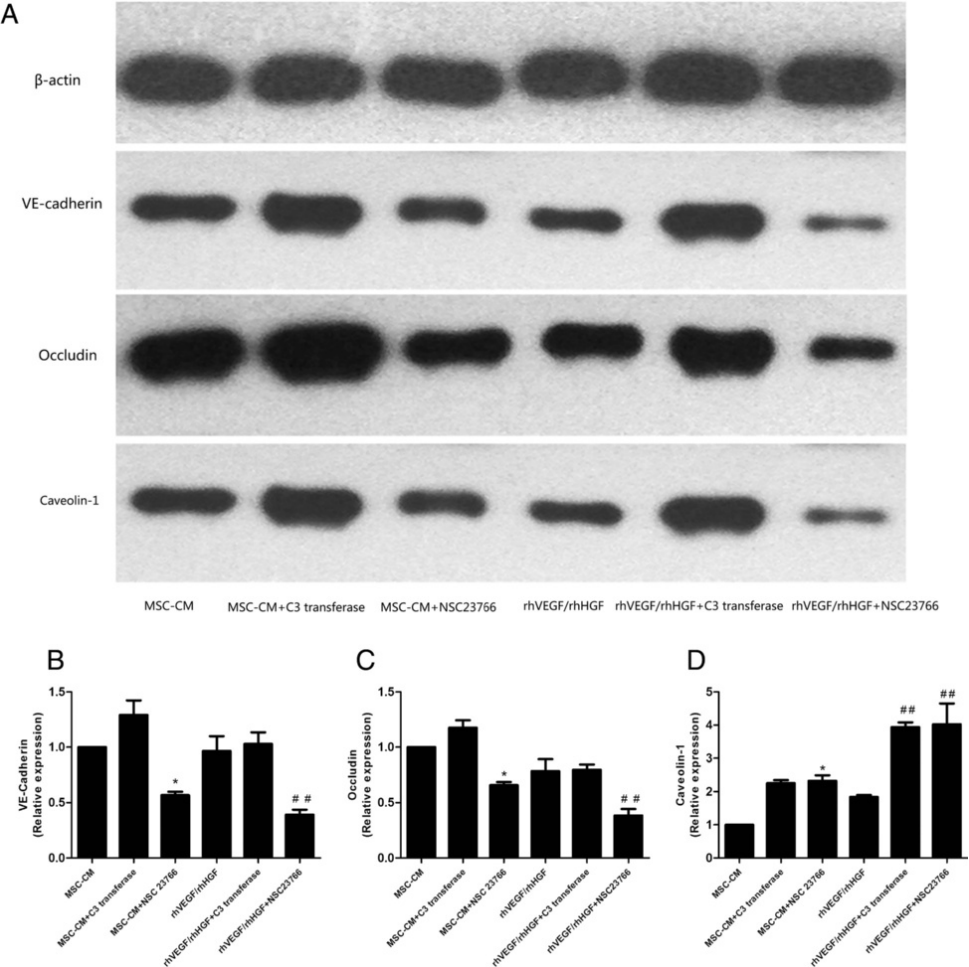
VEGF/HGF and MSC treatments enhanced VE-cadherin and occludin protein expression and reduced caveolin-1 protein expression in LPS-stimulated HPMECs via the RhoA/Rac1 pathway. The results showed that the effects of MSCs and VEGF/HGF on enhancing VE-cadherin (Fig. 10a and b) and occludin protein expression (Fig. 10a and c) were weakened when injured HPMECs were pretreated with the Rac1 inhibitor NSC23766. However, caveolin-1 protein expression (Fig. 10a and d) increased in HPMECs pretreated with the Rac1 inhibitor NSC23766 or with the RhoA inhibitor C3 transferase. n = 3, *p < 0.05; **p < 0.01 vs. MSC group; ^#^ *p* < 0.05 vs. VEGF/HGF group. *CM* Conditioned medium, *HGF* Hepatocyte growth factor, *LPS* Lipopolysaccharide, *MSC* Mesenchymal stem cell, *VEGF* Vascular endothelial growth factor

To determine the detailed mechanism by which MSC and VEGF/HGF treatments use the RhoA/Rac1 pathway to regulate endothelial permeability in injured HPMECs, we examined endothelial VE-cadherin, occludin and caveolin-1 protein expression in HPMECs pretreated with RhoA and Rac1 inhibitors. The results showed that the effects of MSCs and VEGF/HGF on enhancing VE-cadherin and occludin protein expression were weakened when injured HPMECs were pretreated with the Rac1 inhibitor NSC23766 (*p* < 0.05 and *p* < 0.01, respectively; Fig. 10). However, caveolin-1 protein expression increased in HPMECs pretreated with the Rac1 inhibitor NSC23766 or with the RhoA inhibitor C3 transferase (*p* < 0.05 and *p* < 0.01, respectively; Fig. 10). Our research suggested that VEGF/HGF and MSCs enhanced VE-cadherin and occludin protein expression and reduced caveolin-1 protein expression in HPMECs via the RhoA/Rac1 pathway.

## Discussion

ALI is characterised by increased lung permeability, pulmonary oedema and diffuse inflammation, and is involved in alveolar–capillary membrane disruption [13]. MSCs appear to restore endothelial function via a paracrine effect [14]. However, the detailed mechanism by which MSCs improve endothelial permeability remains unclear [15]. In the present study, we found that MSC-secreted HGF protects endothelial barrier function. However, MSC-secreted VEGF synergises with HGF in stabilising EC barrier function. MSC-secreted HGF and VEGF provide the same effects on lessening endothelial paracellular permeability and reducing transcellular permeability. MSC-secreted HGF and VEGF synergistically restore endothelial intercellular AJ remodelling, decrease caveolin-1 protein expression and endothelial apoptosis, and induce EC proliferation. Furthermore, Rac1 is the common pathway by which VEGF and HGF regulate the paracellular and transcellular permeability of ECs.

Endothelial injury results in barrier dysfunction, which contributes to pulmonary oedema in ALI [16]. Two pathways regulate permeability across the endothelial barrier, the paracellular pathway and the transcellular pathway [17, 18]. The primary function of the paracellular pathway is to transport small molecular substances; however, the transcellular pathway is defined as vesicle-mediated transport of macromolecules across the endothelial barrier in a caveolae-dependent manner [19]. In this study, we used an LPS-induced EC permeability injury model. The results showed that the paracellular and transcellular permeability significantly increased at 6 h following LPS stimulation, which is consistent with the results of a previous study [20]. Therefore, reducing transcellular and paracellular permeability must be reduced to maintain endothelial barrier function in ALI.

MSCs potently stabilise endothelium injury by inhibiting endothelial permeability [18, 21]. A strong paracrine capacity has been proposed as the primary mechanism of this function [22]. MSCs are capable of secreting a few factors under normal conditions. To enhance the therapeutic effect of MSCs, some studies have attempted to maximise the paracrine potential of MSCs [23]. For instance, hypoxic preconditioning of MSCs facilitated the release of additional factors [24]. In our study, we designed a hypoxic cell culture model to augment the paracrine potential of MSCs. To investigate the paracrine effect of MSCs on endothelial permeability further, we studied the permeability of HPMECs by co-culture with MSC-CM.

MSC-secreted HGF and VEGF are two important factors associated with endothelial permeability [8, 25, 26]. We found that MSC-secreted HGF significantly decreased endothelial paracellular permeability. Although MSC-secreted VEGF alone did not reduce endothelial paracellular permeability, MSC-secreted VEGF synergises with HGF in contrast to the role of recombinant VEGF [26]. Additionally, MSC-secreted VEGF has similar effects to HGF on increasing occluding protein expression in ECs. The possible explanation is that different quantities and proportions of HGF and VEGF exhibit different effects on endothelial permeability. MSCs secrete VEGF and HGF in proper quantities and proportions, thus playing additive roles in reducing the paracellular permeability of ECs.

The transcellular pathway is defined as the vesicle-mediated transport of macromolecules across the endothelial barrier in a caveolae-dependent manner [27]. Our study showed that LPS increased transcellular endothelial permeability. Similar to a previous study [10], MSC-secreted VEGF reduced the LPS-induced transcellular permeability of ECs. Furthermore, our data indicated that MSC-secreted VEGF synergises with MSC-secreted HGF to reduce the transcellular permeability of ECs. Caveolin-1 regulates the endothelial transcellular transport of macromolecules [28, 29]. The results of this study showed that MSC-secreted HGF and VEGF have an additive effect on reducing caveolin-1 protein expression, which is one of the possible mechanisms by which VEGF and HGF reduce the transcellular permeability of ECs.

An increase in LPS-induced endothelial paracellular permeability is regulated by Rho GTPases. The Rho GTPase Rac1 is required for the maintenance of intercellular adherens and tight junctions, contributing to reducing endothelial paracellular permeability [11, 30]; however, the Rho GTPase RhoA leads to the breakage of intercellular adherens and tight junctions, leading to high paracellular endothelial permeability [31, 32]. Our data showed that MSC-secreted VEGF and HGF reduced paracellular and transcellular endothelial permeability by upregulating Rac1 activity in ECs. The results of our study suggested that Rac1 is the common pathway by which VEGF and HGF regulate the paracellular and transcellular permeability of ECs.

One limitation of our study should be noted. Our study suggested that MSC-secreted VEGF and HGF have an additive role in stabilising EC barrier function and that Rac1 is the common pathway by which MSC-secreted VEGF and HGF regulate the permeability of ECs. However, the mechanism by which MSCs secreted VEGF and HGF is not clearly defined in this study. Our future study will employ further research to verify the detailed mechanism of the additive role between MSC-secreted VEGF and HGF.

## Conclusion

In summary, we demonstrated that MSC-secreted HGF protects endothelial barrier function and that MSC-secreted VEGF may synergize with MSC-secreted HGF to stabilise EC barrier function. MSC-secreted HGF and VEGF may provide synergistic effects that lessen endothelial paracellular permeability and that reduce transcellular permeability. MSC-secreted HGF and VEGF may synergistically restore endothelial intercellular AJ remodelling, decrease caveolin-1 protein expression and endothelial apoptosis, and induce EC proliferation. Furthermore, Rac1 is the common pathway by which VEGF and HGF regulate the paracellular and transcellular permeability of ECs.

## Abbreviations

AJ: Adherens junction
ALI: Acute lung injury
BSA: Bovine serum albumin
CM: Conditioned medium
EC: Endothelial cell
ELISA: Enzyme-linked immunosorbent assay
FITC: Fluorescein isothiocyanate
HGF: Hepatocyte growth factor
hMSC: Human mesenchymal stem cell
HPMEC: Human pulmonary microvascular endothelial cell
LPS: Lipopolysaccharide
MSC: Mesenchymal stem cell
MTT: 3-(4,5-dimethylthiazol-2-yl)-2,5-diphenyltetrazolium
PBS-T: Phosphate-buffered saline-Tween
VEGF: Vascular endothelial growth factor
